# Utility of FOS as diagnostic marker for osteoid osteoma and osteoblastoma

**DOI:** 10.1007/s00428-019-02684-9

**Published:** 2019-11-25

**Authors:** Suk Wai Lam, Arjen H. G. Cleven, Herman M. Kroon, Inge H. Briaire-de Bruijn, Karoly Szuhai, Judith V. M. G. Bovée

**Affiliations:** 1 Department of Pathology, Leiden University Medical Center, Albinusdreef 2, 2333 ZA Leiden, The Netherlands; 2 Department of Radiology, Leiden University Medical Center, Albinusdreef 2, 2333 ZA Leiden, The Netherlands; 3 Department of Cell and Chemical Biology, Leiden University Medical Center, Einthovenweg 20, 2333 ZC Leiden, The Netherlands

**Keywords:** Osteoid osteoma, Osteoblastoma, Immunohistochemistry, Fluorescence in situ hybridization, FOS, Bone tumors

## Abstract

Osteoid osteoma and osteoblastoma are bone-forming tumors shown to harbor *FOS* (87%) and *FOSB* (3%) rearrangements. The aim was to evaluate the immunohistochemical expression of FOS and FOSB in these tumors in comparison to other bone tumors, to evaluate the influence of decalcification, and to correlate immunohistochemical findings with the underlying genetic alteration using fluorescence in situ hybridization (FISH). Immunohistochemistry using whole sections was performed on osteoid osteoma (*n*=23), osteoblastoma (*n*=22), osteoblastoma-like osteosarcoma (*n*=3), reactive (*n*=3), and proliferative (*n*=11) bone lesions. Immunoreactivity in giant cell tumor of bone (*n*=74), aneurysmal bone cyst (*n*=6), chondromyxoid fibroma (*n*=20), osteosarcoma (*n*=85), chondroblastoma (*n*=17), and clear cell chondrosarcoma (*n*=20) was assessed using tissue micro arrays. Strong nuclear expression of FOS in > 50% of the tumor cells was observed in all osteoid osteomas (22/22), in 57% of osteoblastomas (12/21) and in 3/197 control cases. FOS immunoreactivity disappeared after > 3 days decalcification. *FOS* rearrangements were present in 94% of osteoid osteomas and osteoblastomas, with a concordance of 86% between FISH and immunohistochemistry. Two osteoblastomas (5%) were positive for FOSB, as opposed to 8/177 control cases. Additional FISH revealed no *FOSB* rearrangements in these cases. To conclude, in short decalcified biopsies, FOS immunohistochemistry can be used to diagnose osteoid osteoma and osteoblastoma, as overexpression is seen in the majority, being rare in their mimics. FOS immunohistochemistry should not be used after long decalcification. Moreover, low level of focal expression found in other lesions and tissues might cause diagnostic problems, in which case FISH could be employed.

**Electronic supplementary material:**

The online version of this article (10.1007/s00428-019-02684-9) contains supplementary material, which is available to authorized users.

## Introduction

Osteoid osteoma and osteoblastoma are common bone-forming tumors and typically present during the second decade of life. They have no malignant potential, but osteoblastoma can behave locally aggressive [1, 2]. Both lesions are more or less histologically indistinguishable, and distinction is predominantly based on size (diameter below or above 2 cm, respectively) [3]. In addition, osteoid osteomas are usually located in the long bones and present with nocturnal pain relieved by nonsteroidal anti-inflammatory drugs (NSAIDs), while osteoblastomas have a preference for the posterior column of the spine. The most essential feature in osteoid osteoma is the radiographic presence of a central lucent area (nidus), which is surrounded by dense sclerotic bone tissue. In the nidus, regular trabeculae of woven bone are present. These trabeculae are lined by active osteoblasts with vascularized stroma in between. In osteoblastoma, the distribution of woven bone can be slightly less organized, as compared to the nidus of an osteoid osteoma. In the past years, deep sequencing has rapidly advanced the field, as it has provided increased knowledge on the molecular background of bone tumors. Based on these findings, molecular testing as well as specific immunohistochemistry has found its way in routine bone tumor diagnostics that historically heavily relied on morphology and has improved diagnostic accuracy [4, 5]. Recently, recurrent translocations in *FOS* (87%) and *FOSB* (3%) were found in osteoblastoma and osteoid osteoma [6]. Both FOS and FOSB are part of the FOS family of transcription factors and were shown to play a role in diverse biological processes, including osteoblast differentiation and proliferation [7]. Also, similar rearrangements are found in vascular tumors [8–11]. The aim of this study was to evaluate whether the recently found *FOS* and *FOSB* rearrangements can be used as an auxiliary diagnostic tool in routine bone tumor diagnosis. We compared immunohistochemistry of FOS and FOSB between osteoid osteoma and osteoblastoma and other lesions with bone deposition. We evaluated the influence of decalcification and, in addition, correlated the immunohistochemical findings to the underlying genetic alteration using interphase fluorescence in situ hybridization (FISH).

## Materials and methods

### Sample collection

Whole sections were cut from osteoid osteoma (*n*=23) and osteoblastoma (*n*=22). All cases were retrieved from the Department of Pathology at Leiden University Medical Center, with the exception of one osteoblastoma. Since samples were collected from routinely processed diagnostic cases, fixation and decalcification time varied. For all internal cases, samples were decalcified in formic acid for a short period of 4 h or longer (range: 2–15 days) until ready for cutting. For the external cases, exact decalcification time and procedure were unknown. The majority (21/23) of osteoid osteoma samples were decalcified shortly, while for osteoblastoma samples, the decalcification time was more variable.

For comparison we included whole sections of proliferative bone lesions (subungual exostosis (*n*=3), bizarre parosteal osteochondromatous proliferation (BPOP) (*n*=5), and myositis ossificans (*n*=3)), samples with reactive callus formation (*n*=3), and osteoblastoma-like osteosarcoma (*n*=3). Furthermore, sections of previously constructed tissue micro arrays (TMAs) of giant cell tumor of bone (*n*=74) [12], aneurysmal bone cyst (*n*=6) [12], chondromyxoid fibroma (*n*=13) [12], osteosarcoma (*n*=76) [12, 13], chondroblastoma (*n*=11) [12], and clear cell chondrosarcoma (*n*=13) were evaluated [14]. In addition, TMAs of chondroblastoma (*n*=6) and chondromyxoid fibroma (*n*=7) and osteosarcoma (*n*=8) were constructed as described previously [12]. For each sample, three 1.5 mm cores were present on the TMA for representativity. For the osteosarcoma TMA, samples from a previously published cohort were used [15]. For osteosarcoma, both biopsies (*n*=13) as well as resection specimens were used. As far as could be retrieved, among the osteosarcoma cases, there were samples that were not (*n*=13) or short (< 3 days, *n*=19) decalcified.

### Decalcification

A decalcification series of placental tissue was taken along with different decalcification periods, ranging from 4 h to 14 days, using a similar protocol as for internal diagnostic samples. Samples were decalcified using 98–100% formic acid (Merck, Kenilworth, New Jersey, USA), diluted 1:5 in demi water with 2.6% sodium formate.

### Immunohistochemistry

Immunohistochemistry was performed as described previously with minor adjustments [12]. In brief, microwave antigen retrieval was performed in Tris-EDTA (pH 9.0). A rabbit polyclonal antibody was used against the N-terminal region of FOS (clone, F7799, Sigma, St. Louis, Missouri, USA) and a rabbit monoclonal antibody for FOSB (clone, 5G4, Cell Signaling Technology, Danvers, Massachusetts, USA). Sections for FOS staining were pre-incubated with PBS/1% BSA/5% nonfat dry milk for 30 min at room temperature. Primary antibody was diluted in PBS/1% BSA at 1:200,000 for FOS and at 1:30,000 for FOSB, after which slides were incubated overnight at 4 °C. Placenta served as a positive control.

### Evaluation of staining

Slides were scored by two observers independently (SWL and JVMGB). Immunoreactivity was scored according to the intensity of the staining (1 = weak, 2 = moderate, or 3 = strong) and the percentage of tumor cells (i.e., the plump osteoblastic cells lining the osteoid or bone) with nuclear staining (1 = 1–25%, 2 = 26–50%, 3 = 51–75%, and 4 = 76–100%) [16]. Positivity was defined as strong nuclear staining in more than 50% of the tumor cells. For the tumors on the TMAs, an average score of 3 cores was used for analysis.

### Interphase fluorescence in situ hybridization

For in situ hybridization, BAC probes were used proximal and distal to *FOS* and *FOSB*, as described previously [8, 17]. In first instance, a two-color FISH using *FOS* break-apart and *FOSB* break-apart was performed on FFPE sections. In case of no signal due to long decalcification, frozen sections were used as an alternative whenever possible. FISH on FFPE slides and on frozen material was performed as described previously [8, 17, 18]. FISH for *FOS* was performed for all osteoid osteomas and osteoblastomas. In case no *FOS* rearrangement was present, FISH for *FOSB* followed. Also, for the immunohistochemically positive control cases, additional FISH was performed. Slides were scored by two observers independently (SWL, KS).

## Results

### Clinicopathological features of osteoid osteoma and osteoblastoma

Osteoid osteoma and osteoblastoma cases were diagnosed at our institution in a multidisciplinary setting. The average age for osteoid osteoma patients was 22 years (range, 8 to 69 years) and 23 years for osteoblastoma patients (range, 4 to 50 years). In both groups, males were overrepresented (male to female ratio of 3.6:1 and 3.8:1, respectively). For osteoid osteomas, long bones of the lower extremity were affected most (femur, *n*=9; tibia, *n*=3), while seven osteoblastomas (30%) were located in the vertebral column.

**Fig. 1:**
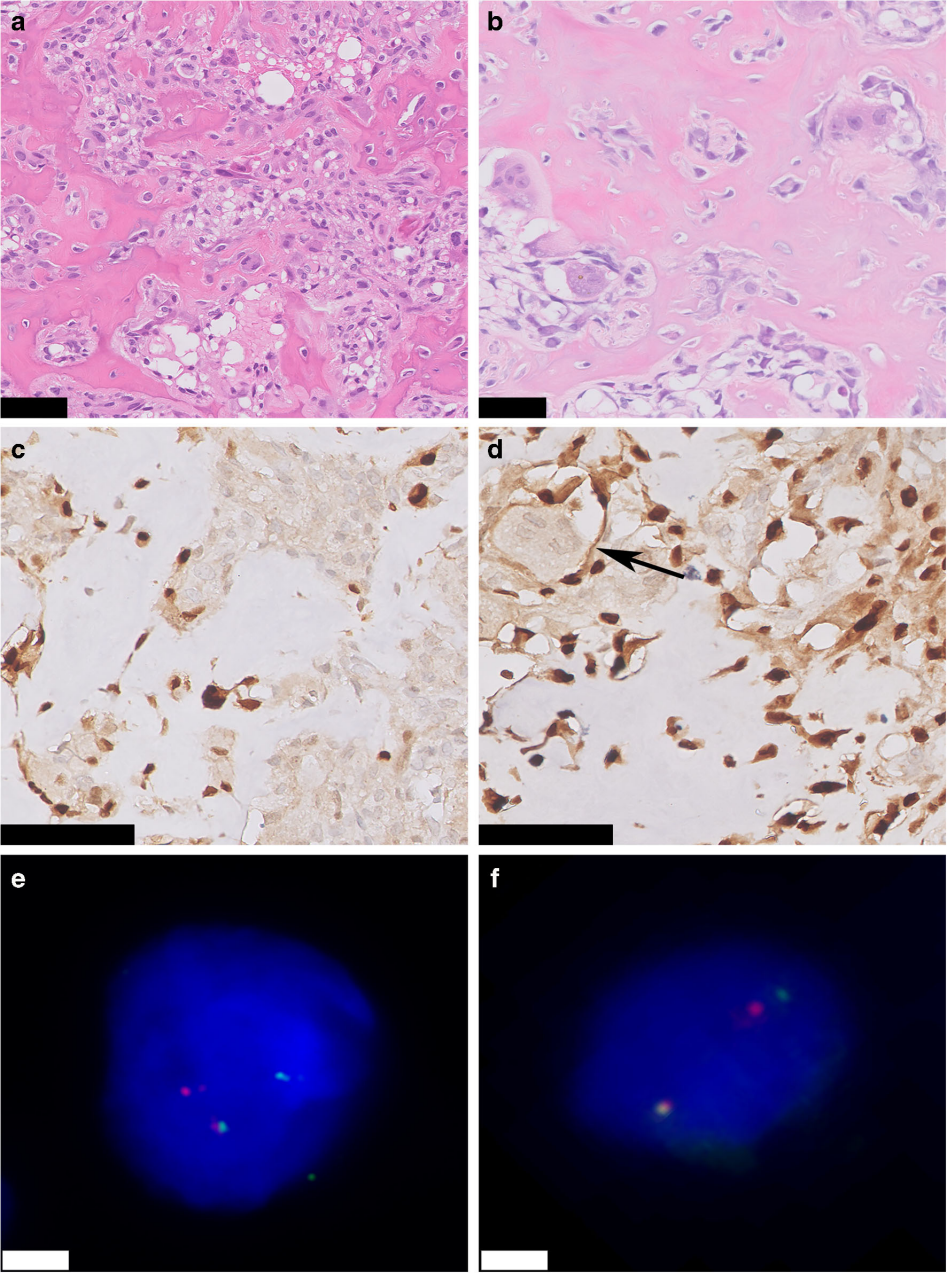
Osteoid osteoma and osteoblastoma. Hematoxylin & Eosin (H&E) staining of the nidus of osteoid osteoma **a** and osteoblastoma **b**. Regular deposited trabeculae of woven bone are surrounded by active osteoblasts. Both osteoid osteoma and osteoblastoma show strong and diffuse nuclear expression of FOS. Osteoclast-like giant cells are negative (arrow) **c** and **d**. Fluorescence in situ hybridization (FISH) using split-apart probes for *FOS* shows a segregated red and green signal in both osteoid osteoma and osteoblastoma, indicating a *FOS* rearrangement **e** and **f**. Scale bar, 50 μm **a–d**. Scale bar, 5 μm **e** and **f**

**Fig. 2:**
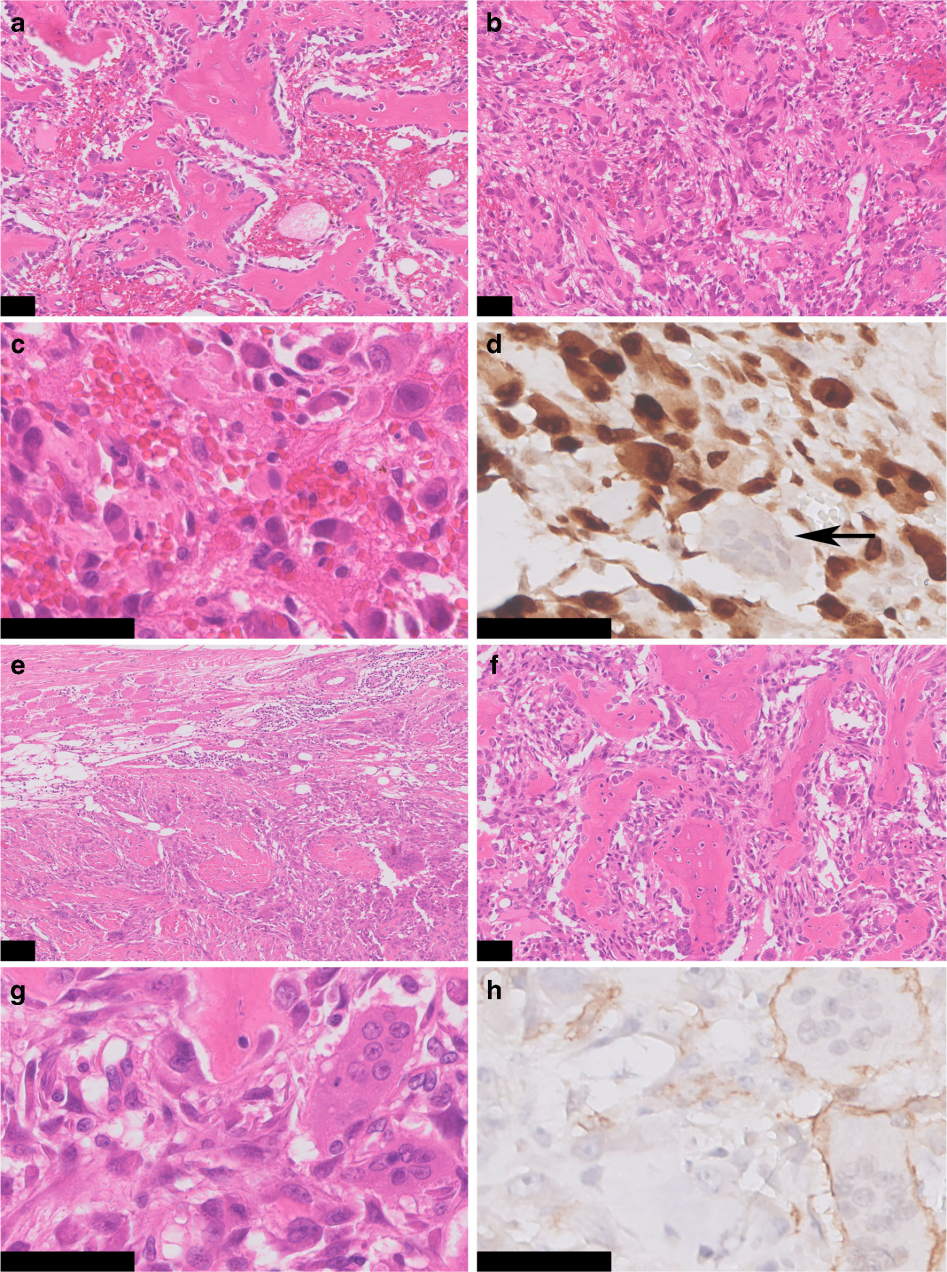
Epithelioid osteoblastoma and osteoblastoma-like osteosarcoma. H&E staining of an epithelioid osteoblastoma shows maturation with presence of trabeculae of woven bone, while the central area shows less osteoid deposition **a** and **b**. Numerous large, plump osteoblasts with abundant eosinophilic cytoplasm are scattered throughout the specimen. Atypia can be frequently encountered, with osteoblasts harboring hyperchromatic and irregular enlarged nuclei, which may resemble osteosarcoma **c**. FOS immunohistochemistry showing diffuse and strong nuclear staining in all osteoblasts. Osteoclasts-like giant cells are negative (arrow) **d**. Osteoblastoma-like osteosarcoma with extensive soft tissue involvement (H&E) **e**. Tumor cells show an epithelioid aspect with enlarged nuclei with a prominent nucleolus. Note the trabeculae of neoplastic woven bone, mimicking osteoblastoma **f** and **g**. FOS immunohistochemistry is negative **h**. Scale bar, 100 μm **a** and **e**. Scale bar, 50 μm **b–d** and **f–h**

All osteoid osteoma specimens showed classic morphology and were composed of trabeculae of woven bone. Surrounding osteoblasts were small, with monomorphic oval nuclei and a moderate amount of cytoplasm. Occasionally, few osteoblasts with more abundant eosinophilic cytoplasm were dispersed throughout the specimen. Osteoclast-like giant cells were present in all cases (Fig. 1). The morphology of osteoblastoma cases was more variable. Three cases showed a clear epithelioid morphology, defined as the presence of numerous large osteoblasts with abundant eosinophilic cytoplasm, in the majority of the specimen. Nuclei were enlarged and were more irregular and hyperchromatic (Fig. 2). Osteoblastoma was distinguished from osteoblastoma-like osteosarcoma, as the latter demonstrated malignant radiological features and presence of infiltrative growth (Fig. 2).

### Immunohistochemistry of FOS and FOSB

**Fig. 3:**
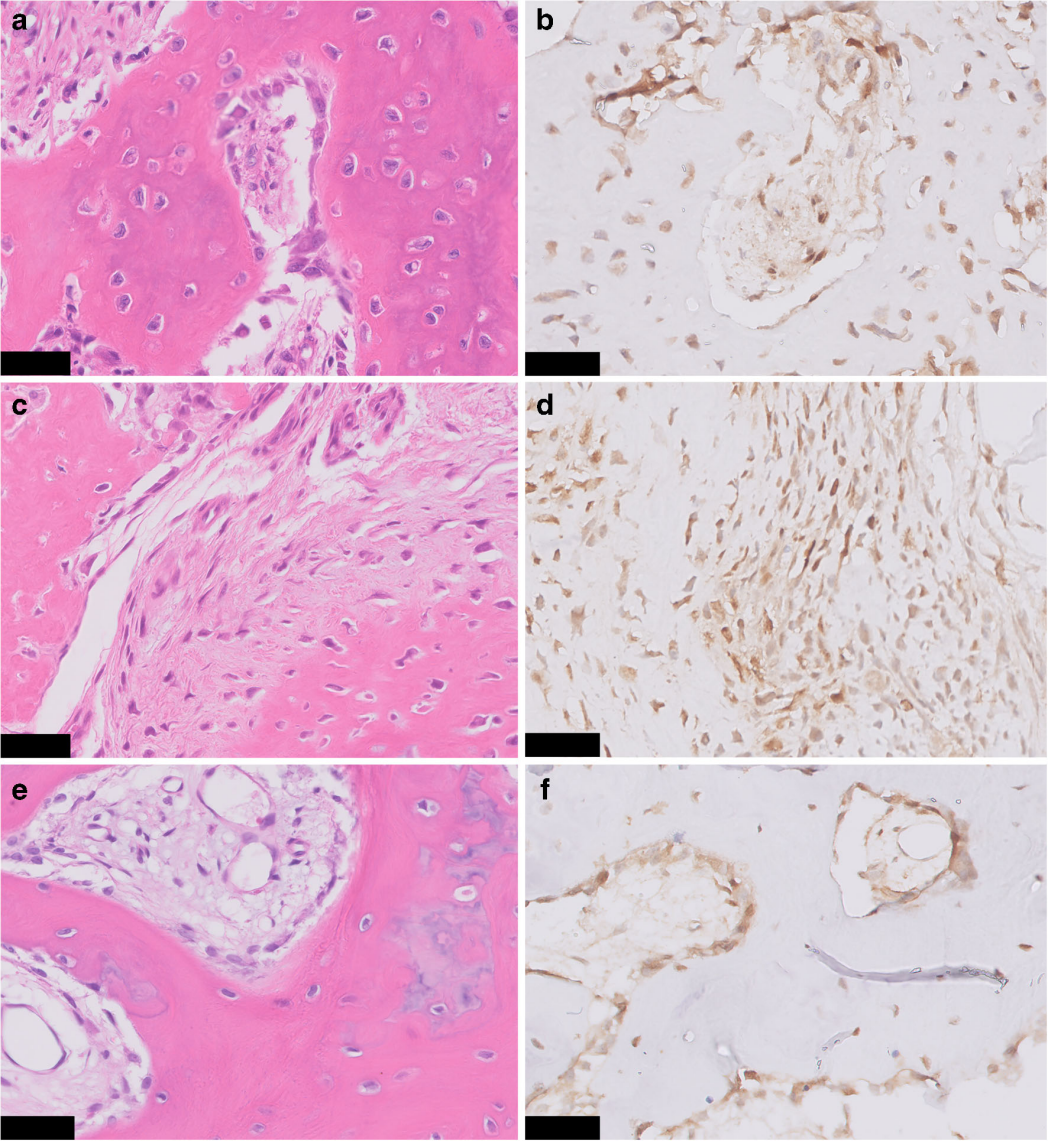
FOS immunohistochemistry in proliferative bone lesions. Myositis ossificans, with peripheral zone showing ill-defined trabeculae of woven bone, rimmed with osteoblasts (H&E) **a**. Immunohistochemistry of FOS showing moderate nuclear staining of osteoblasts **b**. Bizarre parosteal osteochondromatous proliferation with a disorganized mix of woven bone and spindle cells (H&E) **c**, where additional FOS immunohistochemistry shows moderate staining in both components **d**. Central area with trabecular bone in subungual exostosis (H&E) **e**, showing moderate expression of FOS in osteoblasts **f**. Scale bar, 50 μm **(a–f)**

**Fig. 4:**
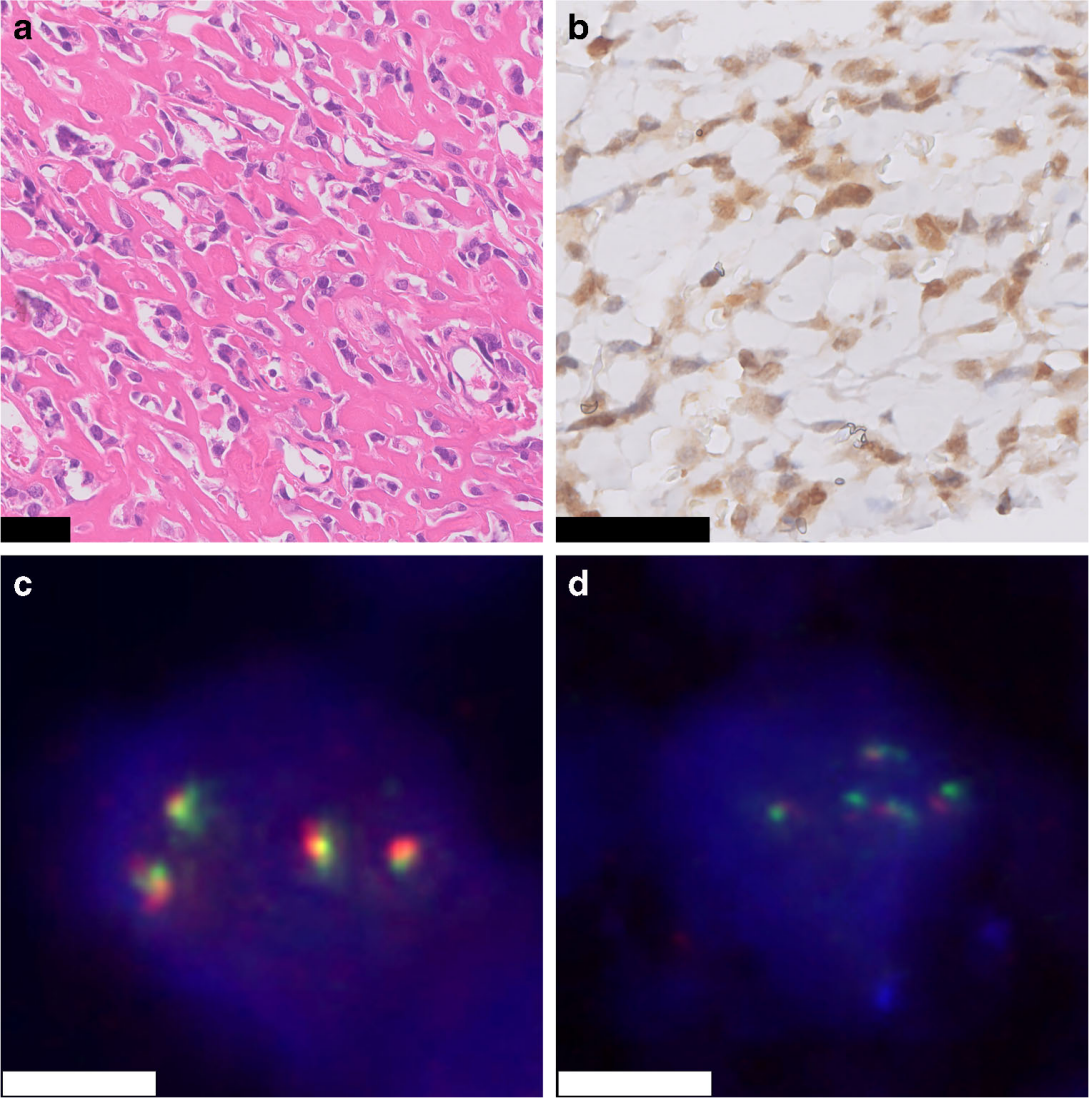
High-grade osteoblastic osteosarcoma. H&E staining shows atypical tumor cells depositing lace-like osteoid (H&E) **(A)**. Immunohistochemistry for FOS shows moderate to strong nuclear staining of the tumor cells **(B)**. Additional FISH for *FOS* and *FOSB* shows gains of the *FOS-* and *FOSB*-locus, respectively **(C and D).** Scale bar, 50 μm **(A and B)**. Scale bar, 5 μm **(C and D)**

Strong and diffuse nuclear staining for FOS was observed in all osteoid osteomas (22/22), in 57% of the osteoblastomas (12/21) (Fig. 1), and in one case with reactive callus formation (1/3). All three osteoblastoma-like osteosarcomas were negative (Fig. 2). Moderate staining in > 50% of the tumor cells was seen in additional 3 osteoblastomas, 1 (out of 54) conventional osteosarcoma, and 1 (out of 6) aneurysmal bone cyst (Supplementary Table 1). Moreover, 45% of the proliferative bone lesions (3/5 BPOP, 1/3 subungual exostosis, and 1/3 myositis ossificans) showed moderate staining in > 50% of the osteoblast-like cells (Fig. 3). Evaluation of 180 samples on TMAs revealed two positive osteosarcomas, which had an osteoblastic and sclerosing morphology (Fig. 4). All other tumors did not show strong and diffuse staining (Table 1). Thus, in total only 3 out of 197 of other bone-forming tumors were positive for FOS immunohistochemistry. The surrounding normal tissues also showed variable moderate to strong nuclear staining, such as endothelial cells and pericytes of mainly larger vessels, striated muscle, chondrocytes, and the epidermis (Supplementary Fig. 1). Osteoclasts were completely negative or showed membranous or cytoplasmic staining.

**Table 1:** Summary of immunohistochemistry for FOS and FOSB

Tumor type	FOS positive* (%)	FOSB positive* (%)
Osteoid osteoma	22/22 (100)	0/22
Osteoblastoma	12/21 (57)	2/21 (10)
Conventional osteosarcoma	2/54 (4)	1/55 (2)
Giant cell tumor of bone	0/73	2/72 (3)
Aneurysmal bone cyst	0/6	0/6
Chondromyxoid fibroma	0/19	0/19
Chondroblastoma	0/14	0/15
Clear cell chondrosarcoma	0/17	-
Reactive bone with callus formation	1/3 (33)	0/2
Proliferative bone lesion:	0/11	5/8 (63)
Subungual exostosis	0/3	-
Bizarre parosteal osteochondromatous proliferation	0/5	4/5 (80)
Myositis ossificans	0/3	1/3 (33)

*defined as strong nuclear expression in > 50% of tumor cells

Two of 21 osteoblastomas were FOSB positive (in addition to FOS), while all 22 osteoid osteomas, all 3 osteoblastoma-like osteosarcomas, and both cases with reactive callus formation were negative. Five proliferative bone lesions (4/5 BPOP and 1/3 myositis ossificans) showed strong and diffuse nuclear staining. Three out of the 164 TMA samples were positive (osteosarcoma (*n*=1) and giant cell tumors of bone (*n*=2)), while the remaining samples were negative (Table 1). Taken together, 8/177 of other bone-forming tumors were FOSB positive.

### Effect of decalcification on FOS and FOSB immunoreactivity

Nuclear staining of decidual cells in placental tissue was moderate to strong in non-decalcified placenta, in short decalcified placenta, and in decalcified placenta up to 3 days. After 3 days, nuclear staining diminished for FOS, turning almost negative after 14 days (Supplementary Fig. 2). For FOSB immunohistochemistry, this phenomenon was not observed as after 14 days of decalcification strong nuclear reactivity was retained (Supplementary Fig. 3).

### Correlation of immunohistochemistry with FISH

Interphase FISH for *FOS* was performed for all osteoid osteomas and osteoblastomas, which was successful in 31/43 cases (27 paraffin, 4 frozen sections). *FOS* rearrangements were present in 94% (*n*=29) of cases, of which 86% (*n*=25) correlated to strong overexpression of FOS at immunohistochemistry (Table 2). Four cases were translocation positive, while immunohistochemistry was scored negative; they displayed moderate staining in a variable percentage of cells, which was below the cutoff we used. All four cases were osteoblastomas, of which decalcification time could not be tracked down or varied between 4 h and 2 days.

**Table 2.** Correlation between FOS immunohistochemistry and interphase fluorescence in situ hybridization in osteoid osteoma and osteoblastoma

FOS	Translocation +	Translocation -
Immunohistochemistry +	25	1
Immunohistochemistry -	4	1

+, positive; -, negative

Two cases lacked *FOS* rearrangement by FISH, while one of these was immunohistochemically positive (Table 2). To rule out cross-reactivity of the FOS antibody, additional FISH for *FOSB* rearrangement was performed, which was negative. After review of H&E slides, clinical records, and radiology, the diagnosis of osteoblastoma remained unchanged (Supplementary Figs. 4 and 5). *FOS* rearrangements were absent in other bone-forming lesions with strong expression of FOS and for which we were able to perform FISH (osteosarcoma (*n*=1) and reactive bone with callus formation (*n*=1)). Of note, the osteosarcoma sample showed multiple copies of the *FOS*-locus (Fig. 4).

In addition, FISH for *FOSB* was performed in 10 cases with strong immunoreactivity for FOSB. FOSB rearrangements were absent in 5 cases in which FISH was successful (BPOP (*n*=1), osteosarcoma (*n*=1), giant cell tumor of bone (*n*=2), and osteoblastoma (*n*=1)). Of these, the osteoblastoma case already showed a *FOS* rearrangement. The osteosarcoma case showed, in addition to multiple copies of the *FOS-*locus, also multiple copies of the *FOSB*-locus (Fig. 4). Both giant cell tumors of bone cases harbored *H3F3A* p.Gly34Trp mutations shown in a previous study [12].

## Discussion

In this study, we evaluated the utility of the use of immunohistochemistry of FOS to diagnose osteoid osteoma and osteoblastoma. So far this has only been tested in small series, with divergent results, as positivity ranged from 0 (*n*=11) to 100% (*n*=3) [6, 19]. Strong overexpression of FOS at immunohistochemistry correlated strongly with the underlying *FOS* rearrangement. While in a previous study in a minority of cases, *FOSB* rearrangements were present, instead of *FOS* rearrangements [6], we did not find any, rendering FOSB immunohistochemistry diagnostically not relevant. Our study indicates that there are two important caveats that pathologists should be aware of when applying immunohistochemistry for FOS to diagnose osteoid osteoma and osteoblastoma.

First, we showed that after > 3 days of acid-based decalcification, immunoreactivity for FOS disappeared. Though decalcification in EDTA preserves DNA and immunogenicity, acid-based solutions are still commonly used and may affect antigen preservation, leading to loss of sensitivity of immunohistochemistry [20]. In this study, a striking difference between osteoid osteoma and osteoblastoma samples for FOS expression was noticed, as all osteoid osteoma, but only 57% of osteoblastomas showed positivity. In general, osteoid osteoma samples were all small and were decalcified for a short period of only 4 h in most cases, as opposed to osteoblastoma samples. The additional decalcified placental series confirmed diminished nuclear staining after a longer period of decalcification for FOS, while FOSB remained intact. Thus, long decalcification times specifically affect FOS immunohistochemistry, and immunohistochemistry should not be used on resection specimens after prolonged acid-based decalcification.

Second, we scored FOS overexpression as strong and diffuse (> 50% of tumor cells) nuclear expression that we found in all 22 osteoid osteomas and in 12 of 21 osteoblastomas. As could be expected based on their role in normal osteoblast maturation and differentiation [7], we noticed moderate to strong nuclear positivity for FOS and FOSB in the areas of bone deposition in several reactive and proliferative bone-forming lesions. Of the neoplasms, only 1 of 6 aneurysmal bone cysts showed moderate staining in > 50% of the tumor cells, while this was absent in other tumors. This can be a pitfall when using immunohistochemistry, necessitating confirmation by FISH under these circumstances. Partial weak staining was noticed in the majority of other samples and should be considered as not representative of translocation-induced overexpression. Moreover, consistent with previous findings in which copy number gains were noticed in FOS immunopositive osteosarcoma [6], we also observed FOS positivity in two osteosarcoma samples (osteoblastic and sclerosing subtype). In one case, FISH was possible, which showed gains of *FOS* and *FOSB*, potentially resulting in overexpression at immunohistochemistry.

The FOS transcription factor family includes FOS, FOSB, FOSL1, and FOSL2 and encodes leucine zipper proteins that can dimerize with proteins of the JUN family, thereby forming the transcription factor complex AP-1. This way, the FOS proteins regulate a diverse array of biological processes, including cell proliferation, differentiation, and survival. Functional studies have shown that FOS and FOSB, together with other family members of FOS family, are highly expressed during normal osteoblast maturation [21]. Retroviral *FOS* oncogene can cause osteosarcoma in mouse model systems, when fused with a highly active promoter and the v-*fos* 3’ untranslated region [22].

Similar rearrangements of *FOS* and *FOSB* were previously found in vascular tumors [8–11]. Identical to *FOS*-rearranged epithelioid hemangioma, the translocations involve various genes or intergenic regions and lead to a premature stop codon, at or early after the break points that always involve exon 4 of *FOS* [6, 8]. This causes loss of the C-terminal end of the protein, rendering the protein resistant to degradation causing high expression in tumor cells [23]. The *FOSB* fusions described in atypical epithelioid hemangioma and pseudomyogenic hemangioendothelioma occur at the N-terminal part of the protein and are most likely induced by promoter swap events, causing upregulation of *FOSB* [10, 11, 24].

For bone tumor pathologists, a challenging diagnostic problem is to discriminate epithelioid osteoblastoma from high-grade osteoblastic osteosarcoma. Epithelioid osteoblastomas can be composed of large, plump osteoblasts, surrounded by abundant eosinophilic cytoplasm. Additional degenerative nuclear atypia can be present, accompanied by mitotic figures. Similarly, osteoblastoma-like osteosarcoma can mimic osteoblastoma. Distinction is of crucial importance, as prognosis and treatment differ significantly. While infiltration of host bone and lack of differentiation towards the periphery seem to be the most discriminating features between (epithelioid) osteoblastoma and (osteoblastoma-like) osteosarcoma, this is not often assessable in biopsy and curettage specimens [25, 26]. Although numbers are small, our present results indicate that immunohistochemistry and/or FISH for FOS can be of help in distinguishing (epithelioid) osteoblastoma from osteosarcoma, especially since there are no specific antibodies or molecular tests for osteosarcoma.

To summarize, FOS immunohistochemistry can be used as an auxiliary tool for osteoid osteoma and osteoblastoma in short decalcified tissue, while FOSB immunohistochemistry is diagnostically not useful. However, FOS immunohistochemistry should not be used after long decalcification, and the low-level focal expression found in other lesions and tissues, especially reactive bone, might be confusing. Under these circumstances, the use of FISH for FOS could be diagnostically useful, for cases where it is difficult to distinguish osteoid osteoma and osteoblastoma from their histologic mimics.

## Electronic supplementary material

Supplementary Fig. 1 FOS immunohistochemistry in normal tissues. Moderate to strong nuclear staining is seen in striated muscle **a,** cartilage **b**, endothelial cells, and smooth muscle cells of medium-sized arteries **c**, and epidermis of the skin **d**. Scale bar, 50 μm a–d (PNG 4728 kb)

High Resolution Image (TIF 12151 kb)

Supplementary Fig. 2 FOS immunohistochemistry on decalcified placental series. Nuclear expression of FOS in decidual cells is seen in non-decalcified placenta **a**. After short decalcification (4 h) **b** and after 3 days of decalcification **c**, nuclear staining remains present. Diminishing staining is seen when decalcified for a longer period of 7 days **d** and almost fully absent after 14 days of decalcification **e**. Scale bar, 50 μm **a–e** (PNG 2130 kb)

High Resolution Image (TIF 8072 kb)

Supplementary Fig. 3 FOSB immunohistochemistry on placental series, including non-decalcified placenta **a**, placenta after short decalcification (4 h) **b**, 3 days **c**, 7 days **d,** and 14 days of decalcification. Strong nuclear expression of FOSB is seen in decidual cells, and expression is not affected even after 14 days of decalcification **e**. Scale bar, 50 μm **a–e** (PNG 2544 kb)

High Resolution Image (TIF 8072 kb)

Supplementary Fig. 4. Axial CT of the left hip. Intracortical lucency on the anterior surface of the femoral neck (arrow) with discrete central mineralization, indicating an osteoid osteoma **a**. H&E slide showing trabeculae of woven bone, rimmed with non-atypical active osteoblasts, compatible with the radiological diagnosis of osteoid osteoma **b.** Immunohistochemistry of FOS shows strong, nuclear staining of active osteoblasts, while FISH showed no *FOS* rearrangement (not shown) **c** (PNG 3729 kb)

High Resolution Image (TIF 12670 kb)

Supplementary Fig. 5 Axial contrast-enhanced T1-weighted MR image. Expansile intracortical lesion arising from the humerus, surrounded by a rim of low signal intensity, representing a bony shell (arrows). Extensive perilesional and peritumoral edema of the soft tissues is present (asterisks) **a**. Axial CT image in bone setting. Expansile intracortical lesion surrounded by a thin bony shell (arrows) arising from the humerus. Together with the MR image, the appearance is very suggestive of an osteoblastoma **b**. H&E staining shows regular deposition of trabeculae of woven bone, surrounded by active osteoblasts, compatible with the radiological diagnosis of osteoblastoma **c**. Immunohistochemistry for FOS shows only weak to moderate nuclear staining, after 10 days of decalcification. Additional FISH showed no *FOS* rearrangement (not shown) **d** (PNG 3013 kb)

High Resolution Image (TIF 12213 kb)

Supp Table 1 (DOCX 14 kb)
