# Supplementary figures and images for: Utility of FOS as diagnostic marker for osteoid osteoma and osteoblastoma
Source: Virchows Arch. 2019 Nov 25;476(3):455–63. doi: 10.1007/s00428-019-02684-9 (PMC7085481; doi:10.1007/s00428-019-02684-9)

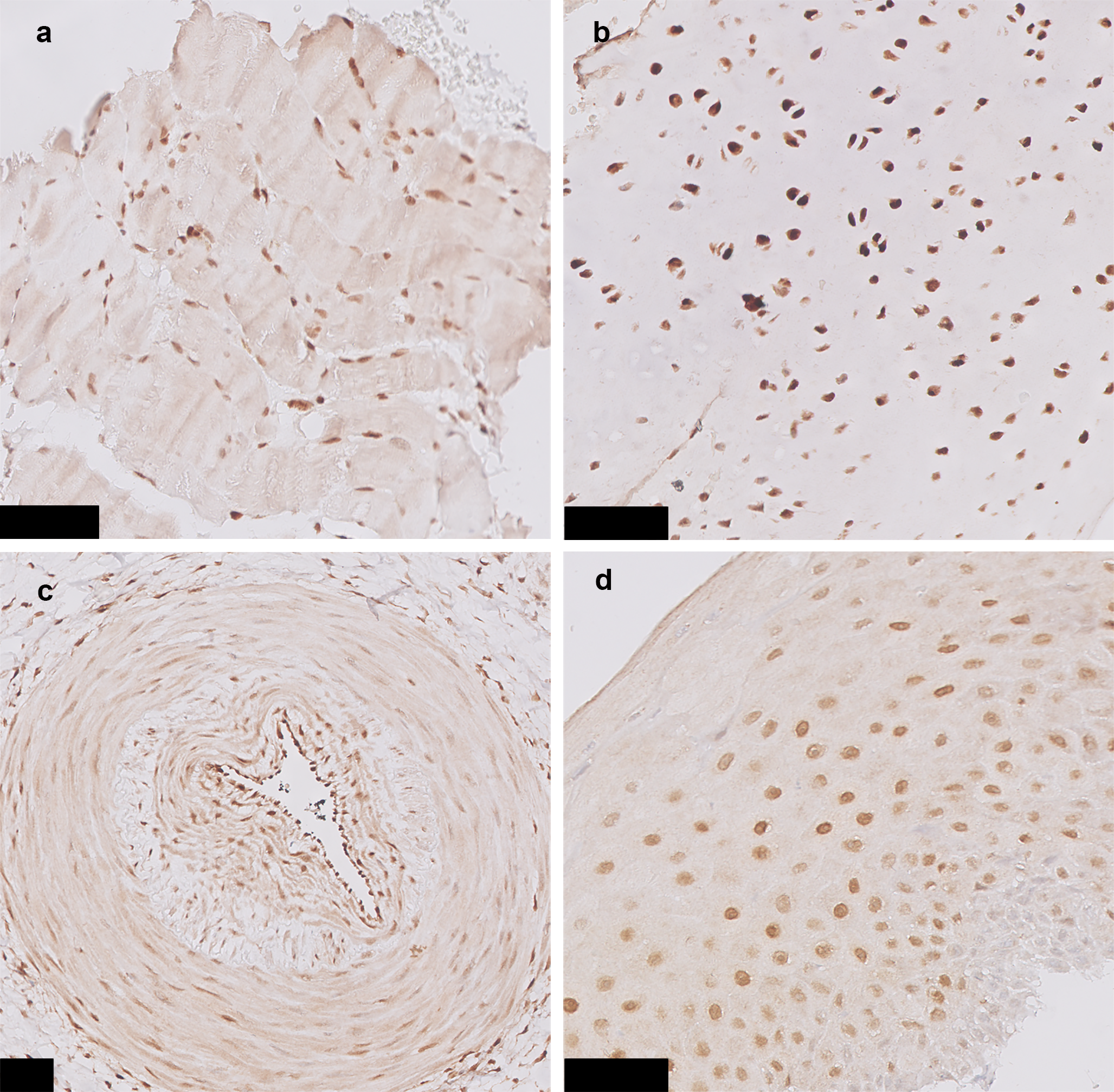

Supplement: Supplementary file 1 — FOS immunohistochemistry in normal tissues. Moderate to strong nuclear staining is seen in striated muscle a, cartilage b, endothelial cells, and smooth muscle cells of medium-sized arteries c, and epidermis of the skin d. Scale bar, 50 μm a–d (PNG 4728 kb) [file 428_2019_2684_Fig5_ESM.png]

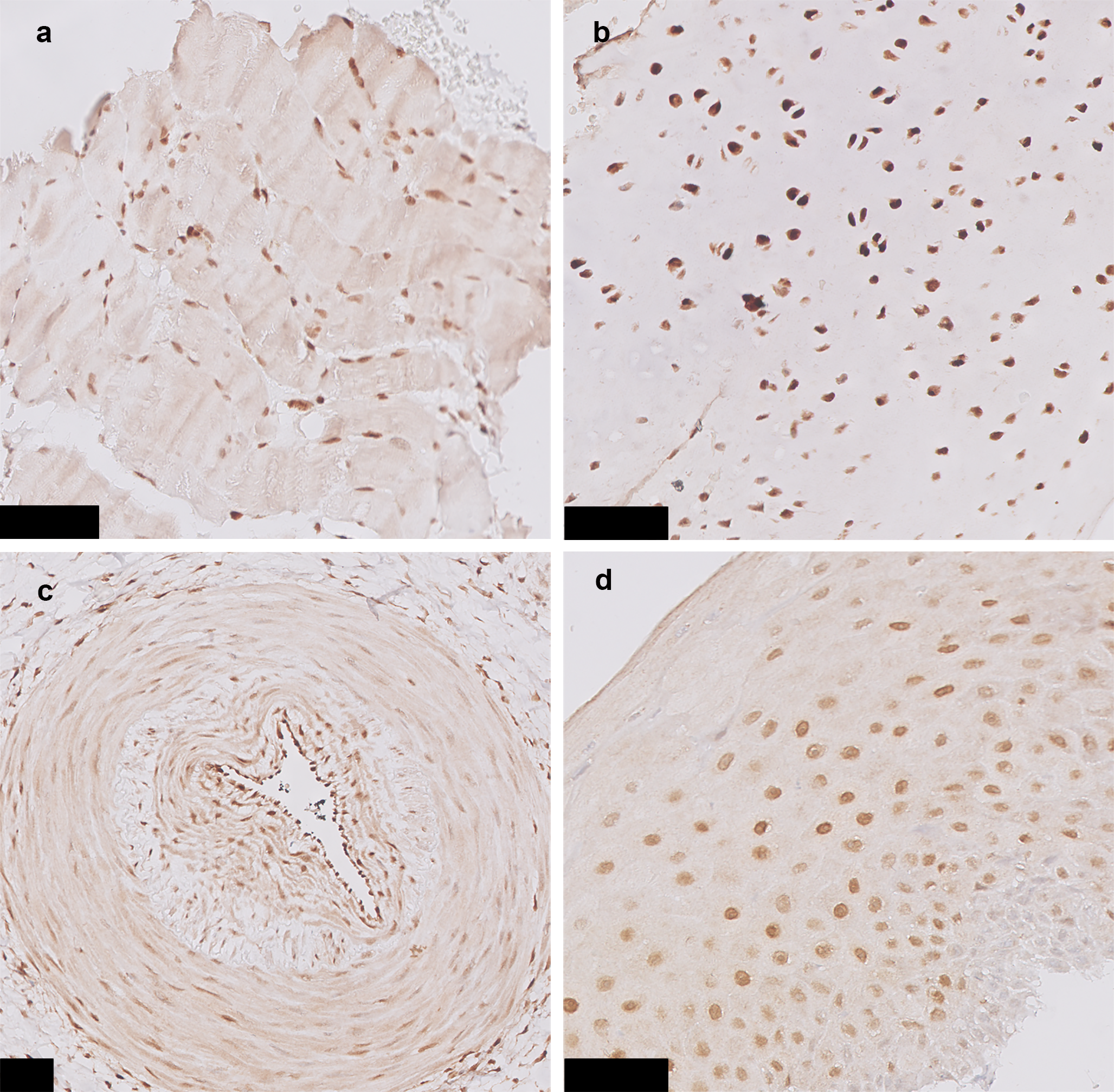

Supplement: Supplementary file 2 — High Resolution Image (TIF 12151 kb) [file 428_2019_2684_MOESM1_ESM.tif]

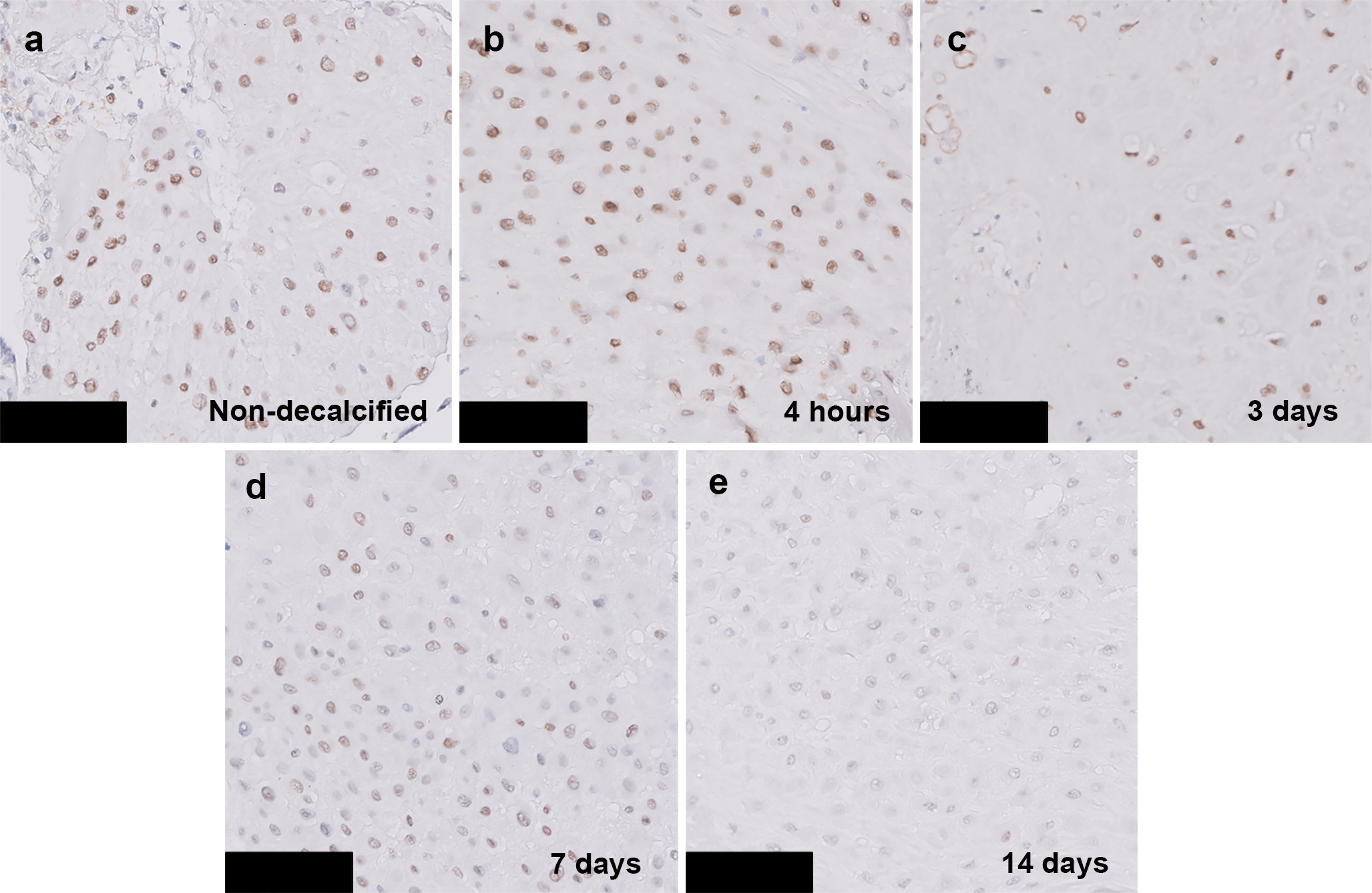

Supplement: Supplementary file 3 — FOS immunohistochemistry on decalcified placental series. Nuclear expression of FOS in decidual cells is seen in non-decalcified placenta a. After short decalcification (4 h) b and after 3 days of decalcification c, nuclear staining remains present. Diminishing staining is seen when decalcified for a longer period of 7 days d and almost fully absent after 14 days of decalcification e. Scale bar, 50 μm a–e (PNG 2130 kb) [file 428_2019_2684_Fig6_ESM.png]

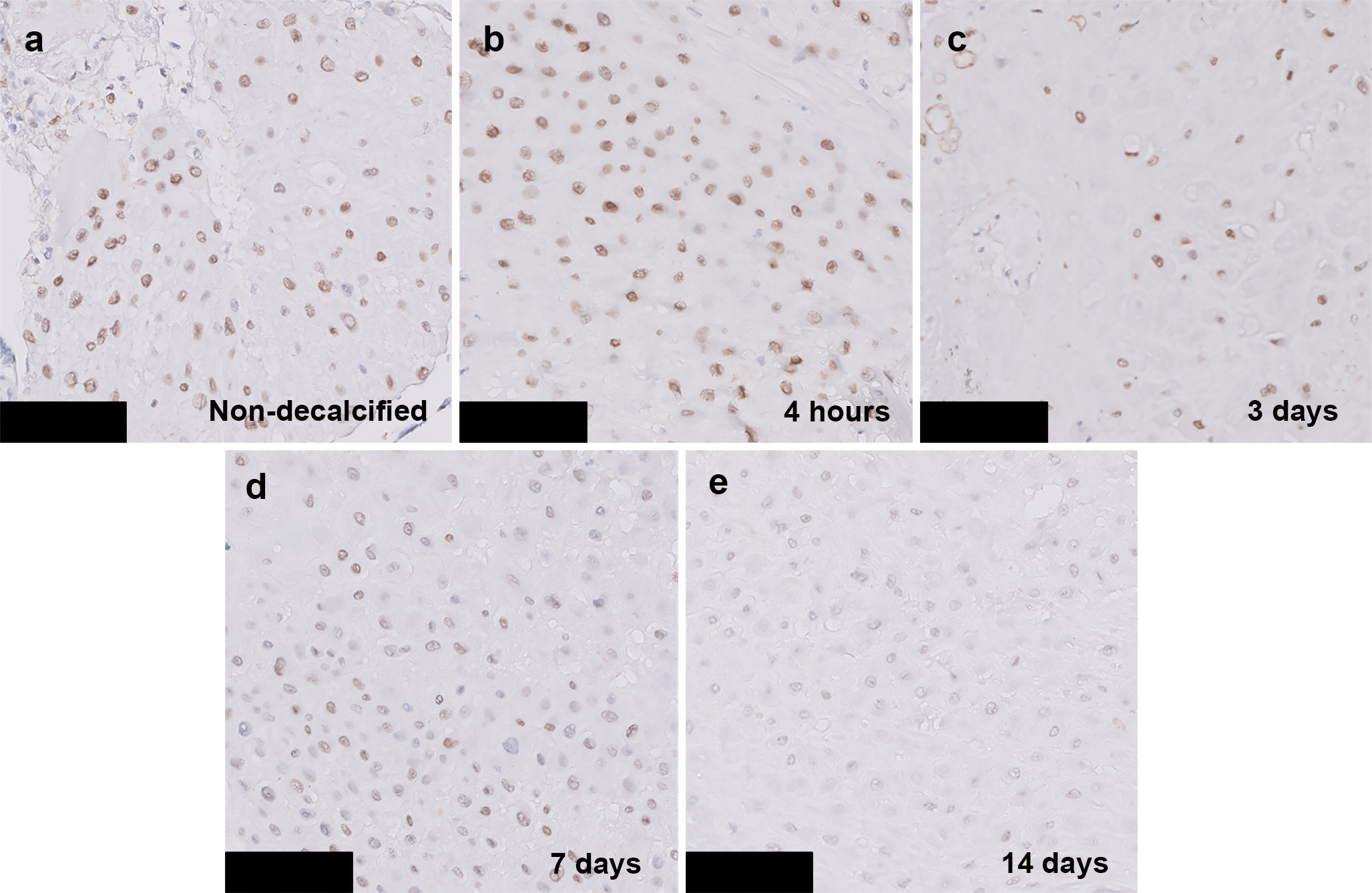

Supplement: Supplementary file 4 — High Resolution Image (TIF 8072 kb) [file 428_2019_2684_MOESM2_ESM.tif]

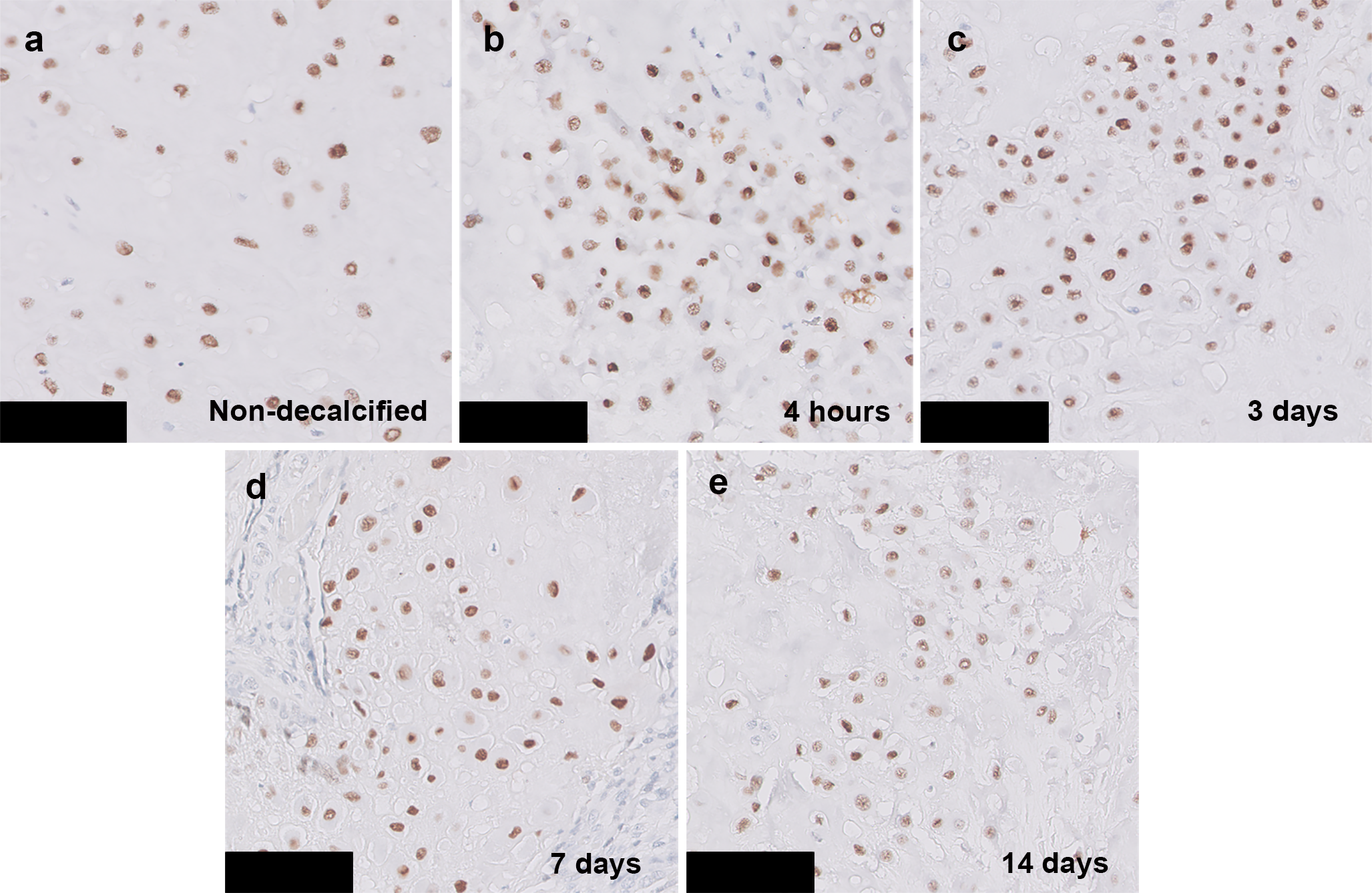

Supplement: Supplementary file 5 — FOSB immunohistochemistry on placental series, including non-decalcified placenta a, placenta after short decalcification (4 h) b, 3 days c, 7 days d, and 14 days of decalcification. Strong nuclear expression of FOSB is seen in decidual cells, and expression is not affected even after 14 days of decalcification e. Scale bar, 50 μm a–e (PNG 2544 kb) [file 428_2019_2684_Fig7_ESM.png]

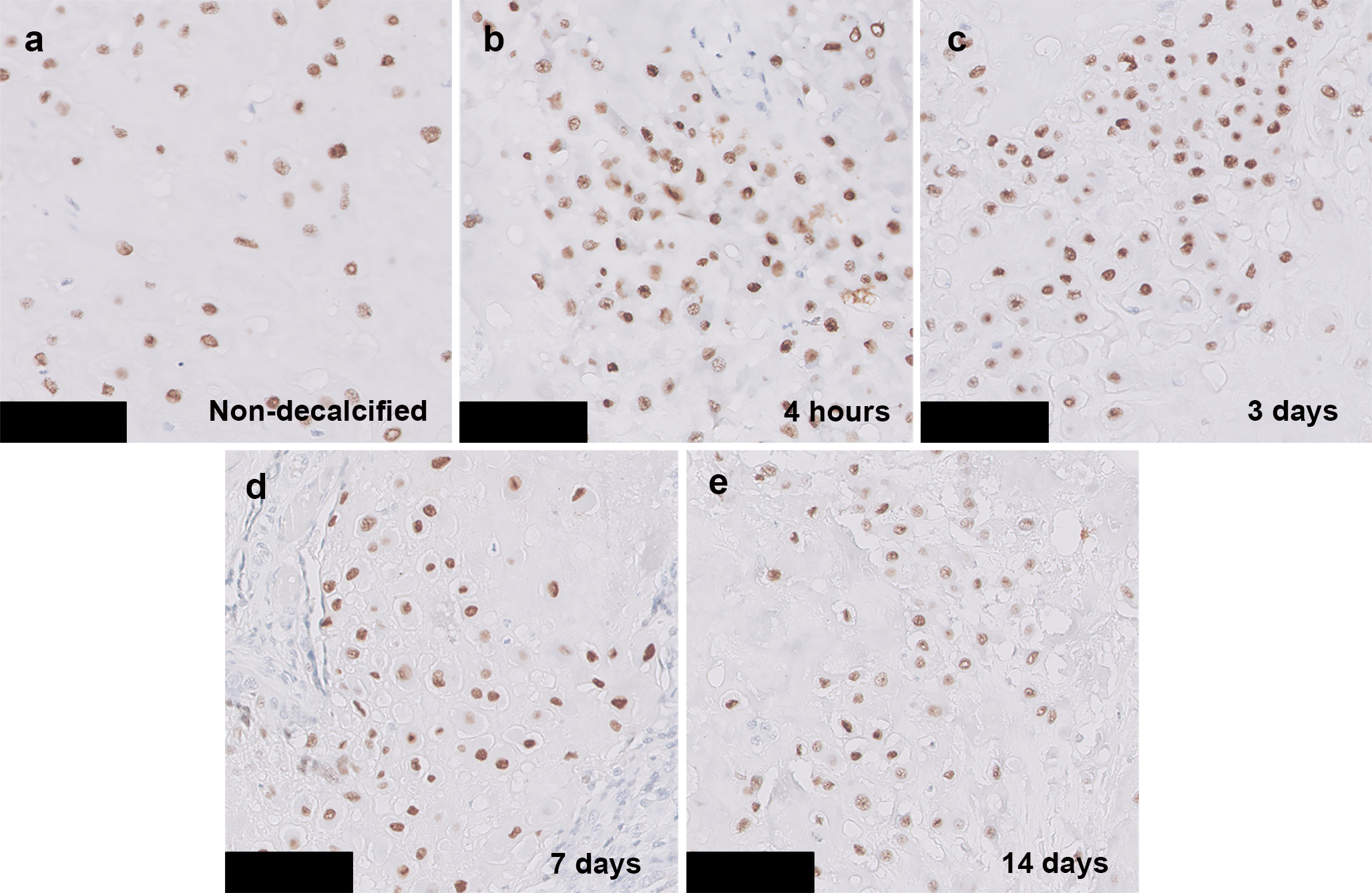

Supplement: Supplementary file 6 — High Resolution Image (TIF 8072 kb) [file 428_2019_2684_MOESM3_ESM.tif]

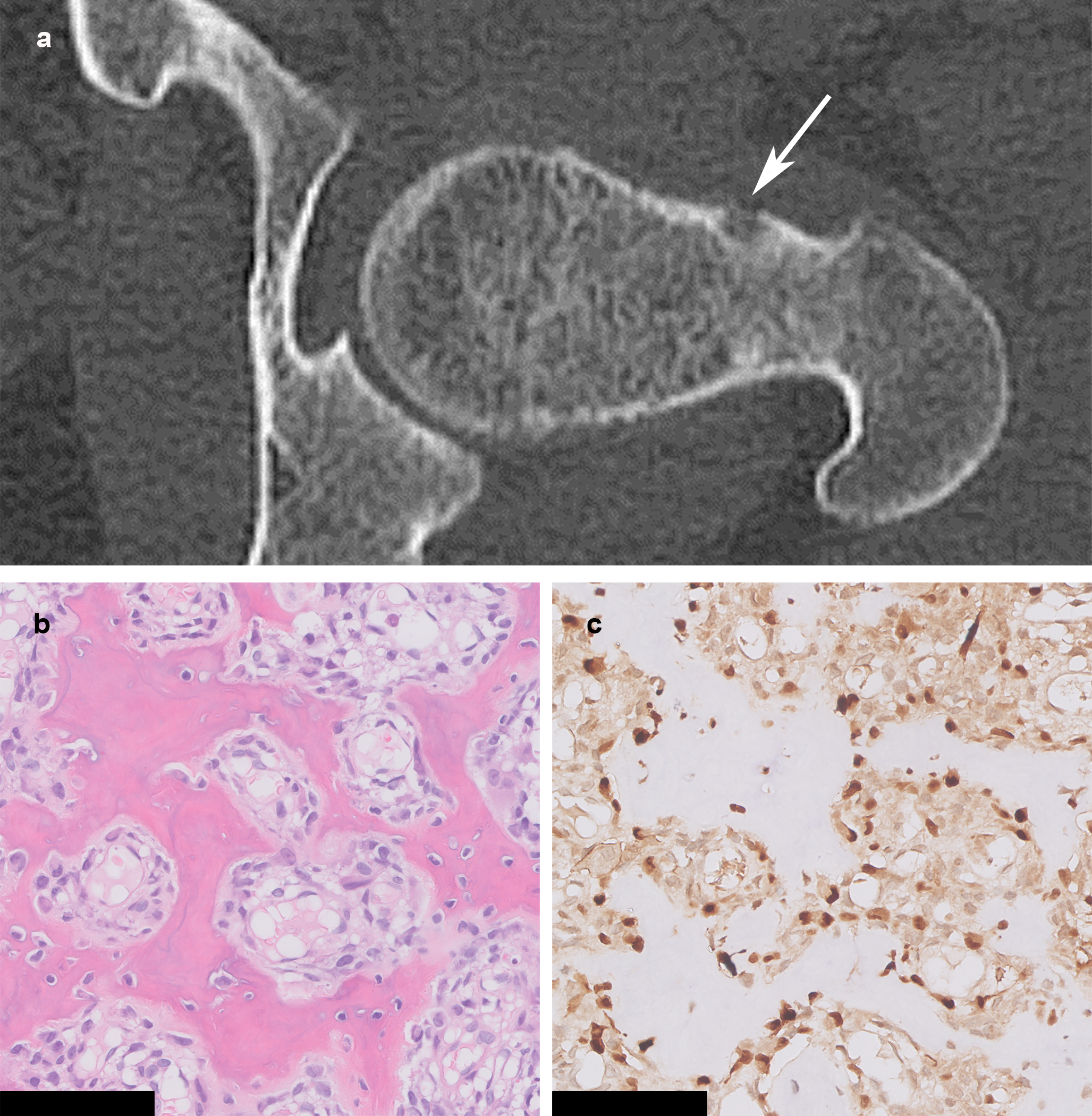

Supplement: Supplementary file 7 — Axial CT of the left hip. Intracortical lucency on the anterior surface of the femoral neck (arrow) with discrete central mineralization, indicating an osteoid osteoma a. H&E slide showing trabeculae of woven bone, rimmed with non-atypical active osteoblasts, compatible with the radiological diagnosis of osteoid osteoma b. Immunohistochemistry of FOS shows strong, nuclear staining of active osteoblasts, while FISH showed no FOS rearrangement (not shown) c (PNG 3729 kb) [file 428_2019_2684_Fig8_ESM.png]

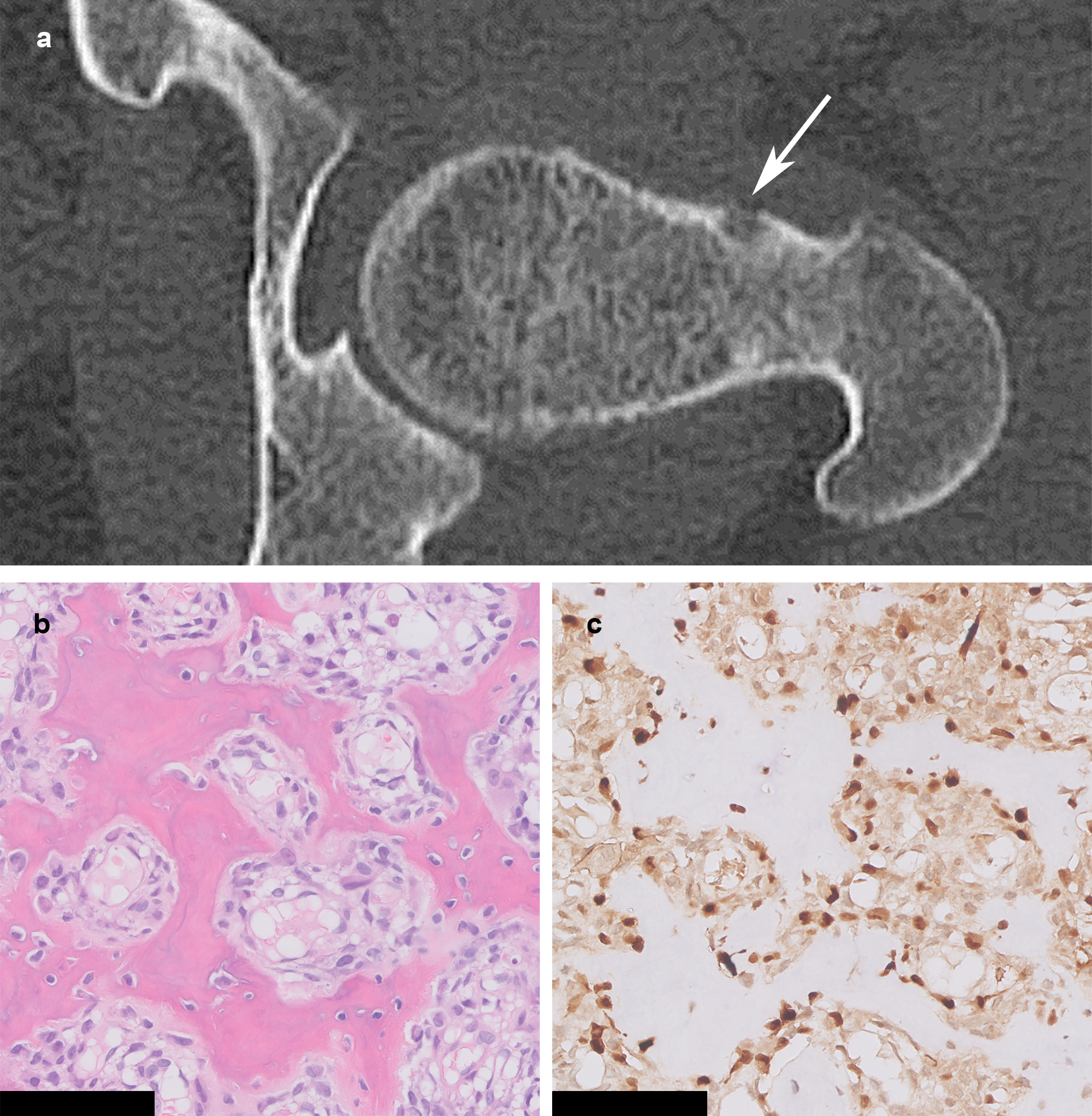

Supplement: Supplementary file 8 — High Resolution Image (TIF 12670 kb) [file 428_2019_2684_MOESM4_ESM.tif]

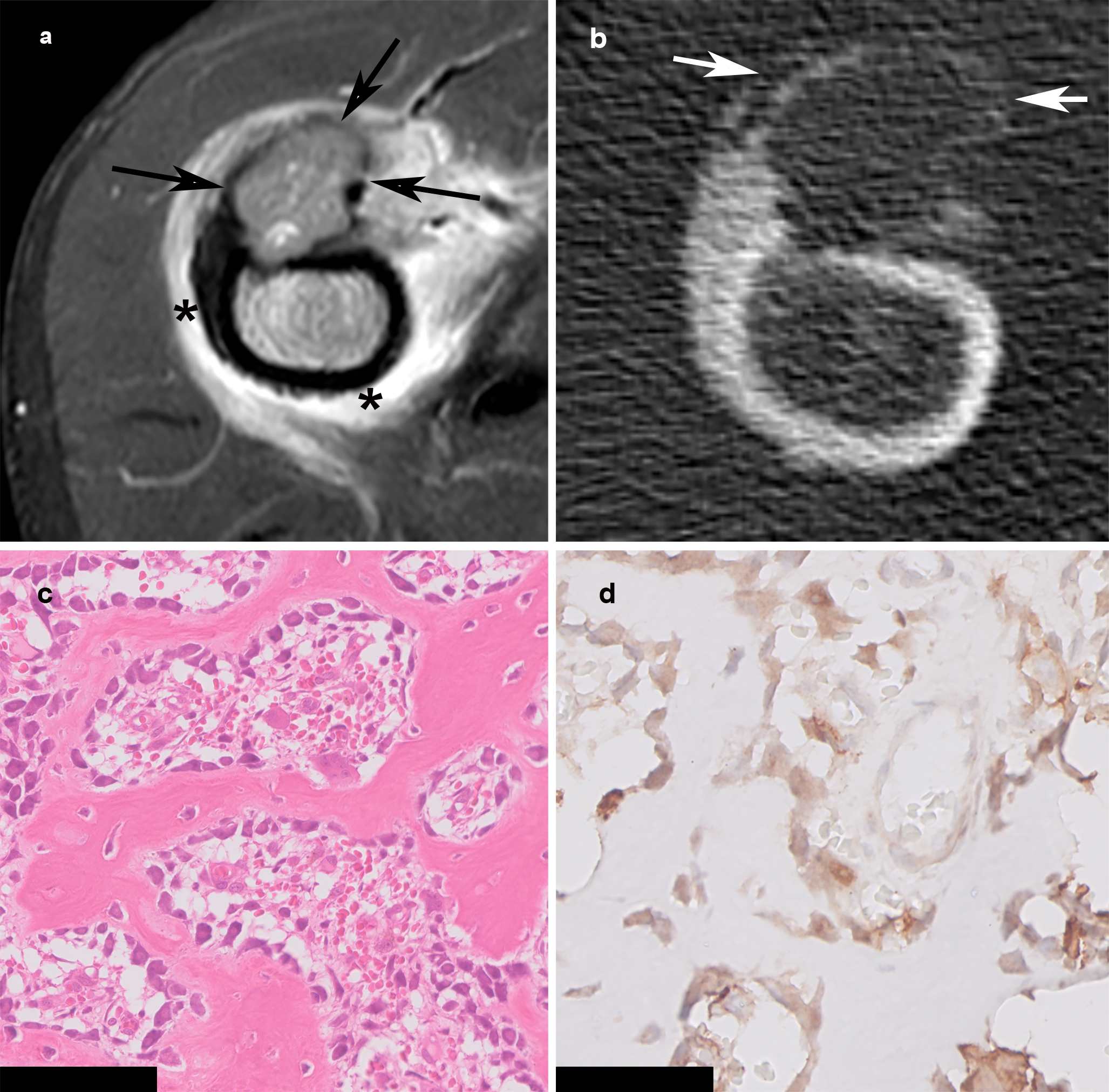

Supplement: Supplementary file 9 — Axial contrast-enhanced T1-weighted MR image. Expansile intracortical lesion arising from the humerus, surrounded by a rim of low signal intensity, representing a bony shell (arrows). Extensive perilesional and peritumoral edema of the soft tissues is present (asterisks) a. Axial CT image in bone setting. Expansile intracortical lesion surrounded by a thin bony shell (arrows) arising from the humerus. Together with the MR image, the appearance is very suggestive of an osteoblastoma b. H&E staining shows regular deposition of trabeculae of woven bone, surrounded by active osteoblasts, compatible with the radiological diagnosis of osteoblastoma c. Immunohistochemistry for FOS shows only weak to moderate nuclear staining, after 10 days of decalcification. Additional FISH showed no FOS rearrangement (not shown) d (PNG 3013 kb) [file 428_2019_2684_Fig9_ESM.png]

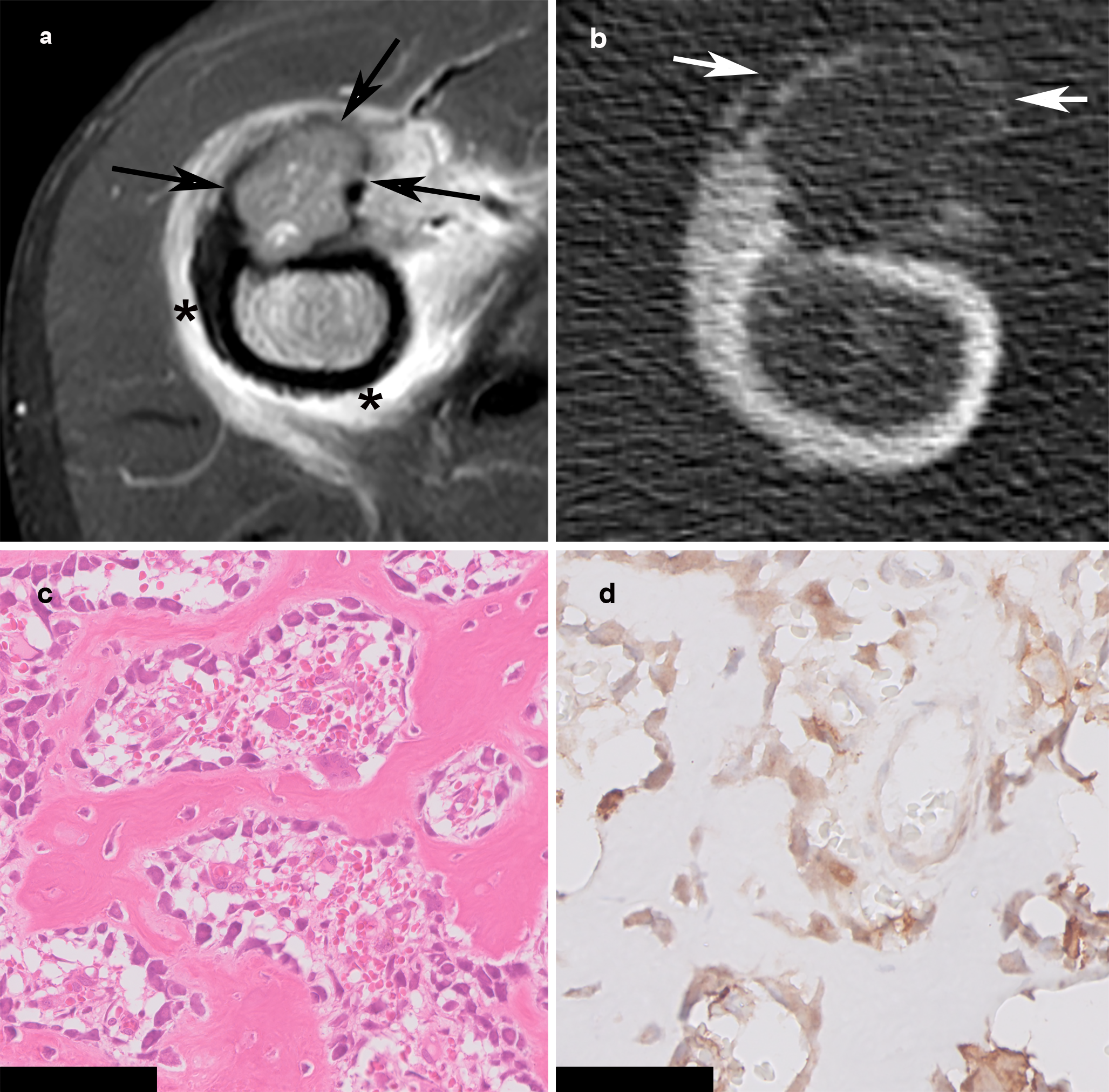

Supplement: Supplementary file 10 — High Resolution Image (TIF 12213 kb) [file 428_2019_2684_MOESM5_ESM.tif]
