# Supplementary material for: Utility of FOS as diagnostic marker for osteoid osteoma and osteoblastoma
Source: Virchows Arch. 2019 Nov 25;476(3):455–63. doi: 10.1007/s00428-019-02684-9 (PMC7085481; doi:10.1007/s00428-019-02684-9)
Supplement: Supplementary file 11 — (DOCX 14 kb) [file 428_2019_2684_MOESM6_ESM.docx]

Supplementary Table 1. Summary of immunohistochemistry for FOS with moderate staining in >50% of tumour cells

| Tumour type | Cases (%) |  |
| --- | --- | --- |
| **Osteoid osteoma** | 0/22 |  |
| **Osteoblastoma** | 3/21 (14) |  |
| **Conventional osteosarcoma** | 1/54 (2) |  |
| **Giant cell tumour of bone** | 0/73 |  |
| **Aneurysmal bone cyst** | 1/6 (17) |  |
| **Chondromyxoid fibroma** | 0/19 |  |
| **Chondroblastoma** | 0/14 |  |
| **Clear cell chondrosarcoma** | 0/17 |  |
| **Reactive bone with callus formation** | 0/3 |  |
| **Proliferative bone lesion:** | 5/11 (45) |  |
| - Subungual exostosis | 1/3 |  |
| - Bizarre parosteal osteochondromatous proliferation | 3/5 |  |
| - Myositis ossificans | 1/3 |  |
